# A novel technique for isolating DNA from Tempus™ blood RNA tubes after RNA isolation

**DOI:** 10.1186/s13104-018-3671-4

**Published:** 2018-08-06

**Authors:** Jason A. Ferrante, Michelle R. Giles, Emily Benzie, Margaret E. Hunter

**Affiliations:** 1 Cherokee Nation Technologies contracted to the U.S. Geological Survey, Wetland and Aquatic Research Center, 7920 NW 71st Street, Gainesville, FL 32653 USA; 2 U.S. Geological Survey, Wetland and Aquatic Research Center, 7920 NW 71st Street, Gainesville, FL 32653 USA; 3 University of California-Davis, 1 Shields Ave, Davis, CA 95616 USA

**Keywords:** Blood, Tempus, RNA, Genomic DNA, Isolation, Preservation, Wildlife

## Abstract

**Objective:**

We use Tempus blood RNA tubes (Applied Biosystems) during health assessments of American moose (*Alces alces* spp.) as a minimally invasive means to obtain RNA. Here we describe a novel protocol to additionally isolate high-quality DNA from the supernatant remaining after the RNA isolation methodology. Metrics used to qualify DNA quality included measuring the concentration, obtaining a DNA integrity number from a genomic DNA ScreenTape assay (Agilent), and running the isolated DNA on an agarose gel.

**Results:**

Of the 23 samples analyzed, the average DNA concentration was 121 ng/µl (range 4–337 ng/µl) and a genomic DNA ScreenTape assay of seven samples indicated high DNA integrity values for 6 of the 7 samples (range 9.1–9.4 out of 10). Of the DNA sent for genotyping by sequencing, all proved to be of sufficient integrity to yield high-quality next-generation sequence results. We recommend this simple procedure to maximize the yield of both RNA and DNA from blood samples.

## Introduction

High-quality genetic and genomic material is an important resource for wildlife studies [1–4]. Specifically, DNA often used for genome sequencing, parentage studies and individual identification, as well as for investigating population structure and speciation [5–7]. Acquiring RNA during field efforts facilitates animal health and disease research, as RNA can be used to identify viruses as well as to quantify the transcription level of genes impacted by environmental or physiological stressors [1, 8, 9]. The collection and preservation of RNA, which is particularly susceptible to degradation, has been limited in the past by the ability to isolate or freeze the sample quickly.

The preservation of genetic and genomic material in the field during wildlife health assessments can be limited by access to laboratory resources and refrigeration, especially when scientists work in remote locations or challenging conditions. Therefore, rapid preservation of RNA and DNA during wildlife health assessments can greatly improve the robustness of genetic studies. Liquid preservation buffers have been developed which denature enzymes and protect genetic material from degradation [10–12]. Some of these buffers even allow for preservation of nucleotides in whole blood at room temperature for days or weeks [13–15]. Once drawn, researchers have time to transport the blood samples to the laboratory without refrigeration for subsequent processing and long-term storage.

For our research, we collect blood by using Tempus blood RNA tubes (Applied Biosystems) as they facilitate opportunistic, minimally invasive blood sampling from our species of interest, the American moose (*Alces alces* spp.). The Tempus tubes are vacutainer blood tubes containing a guanidine hydrochloride solution which immediately lyses blood cells and stabilizes RNA and DNA nucleotides, while inactivating cellular RNAses and DNAses. This direct drawing of the blood into the stabilizing solution also limits the potential for contamination incurred through traditional methods of sample transfer and processing. The Tempus protocol selectively precipitates and isolates RNA, while proteins and genomic DNA (gDNA) remain in solution. Generally, a separate sample of whole blood or a tissue section would be used to obtain gDNA.

To limit undue stress to the animal during health assessments, sample collection must be completed efficiently with minimal sample volumes. Further, costs are curtailed by limiting the number of samples to process downstream. In this study, moose health assessments are frequently conducted by helicopter, charged by the hour, and in remote locations. Therefore, we aimed to decrease collection and processing time, while increasing our sample material acquisition. To streamline our sample collection, we developed and tested a straightforward protocol for gDNA isolation from Tempus blood tubes following RNA isolation. To meet our sample yield criteria, the protocol needed to produce DNA in high enough quantities and quality for genome-wide genotyping by sequencing analyses.

## Main text

### Materials and methods

#### Sample collection

Whole blood was collected from 23 moose during health assessments. Approximately 3 ml of blood was dispensed directly into each Tempus RNA blood tube. The tubes were shaken vigorously for 10 s to homogenize the blood with the stabilizing agent (6 ml of a guanidine hydrochloride solution). The samples were stored at room temperature and then refrigerated at 4 °C as soon as possible (from immediately to 1 day later). Within 1–5 days after collection, the tubes were shipped overnight on ice to the laboratory and immediately frozen at − 20 °C for up to 3 weeks before processing. The Tempus tubes stabilize RNA (and presumably DNA) for up to 5 days at room temperature, at least 1 week at 4 °C and 1.5 years frozen at − 80 °C.

#### RNA isolation

Prior to DNA isolation, RNA was isolated following manufacturers protocols [16]. The protocol begins with combining the thawed Tempus tube sample with 3 ml 1× phosphate buffered serum (PBS) in a 50 ml conical tube, vortexing for 30 s, and centrifuging at 3000×*g* in 4 °C for 30 min. This allowed the RNA to pellet on the bottom of the conical tube. The supernatant was then retrieved into 15 ml conical tubes and stored for DNA isolation at − 20 °C while the RNA pellet was further processed following the published protocol.

#### DNA isolation

We were unable to find literature describing how to effectively recover DNA from the supernatant after the RNA isolation step, so hereafter we share our developed protocol. The retrieved supernatant contained the remainder of the blood sample (lacking RNA) which included gDNA, denatured protein, the stabilizing solution, and PBS. The DNA was extracted from the supernatant using a modified phenol:chloroform:isoamyl alcohol (PCI) DNA isolation protocol [17]. Briefly, we transferred 1 ml of the supernatant to a five ml microcentrifuge tube and added 1.5 ml of sodium chloride–tris–EDTA buffer (STE). A volume of 2.1 ml of PCI (25:24:1, Amaresco) was added to the supernatant and STE, shaken gently, and allowed to sit for 5 min. The tubes were then centrifuged at room temperature for 5 min at 12,000×*g*. The aqueous layer (supernatant) at the top was transferred to a new 5 ml tube, being especially careful not to include any of the CI layer below, and the previous PCI step was repeated. This process was then performed with 2.1 ml of chloroform isoamyl alcohol (CI; 24:1; Amaresco), but with agitation every minute during the 5 min sitting period. The samples were centrifuged at room temperature for 5 min at 12,000×*g* and the aqueous layer was again transferred to a new 5 ml microcentrifuge tube. In this tube, the CI step was repeated to clean the large samples containing proteins. The CI step does not necessarily need to be repeated if samples are small and clean from residue. After the second CI step, the aqueous phase was again transferred to a new 5 ml tube and 150 µl of 3 M sodium acetate was added. The tube was topped off to the 5 ml line with − 20 °C 95% ethanol, well shaken, and left in the freezer (− 20 °C) overnight to precipitate.

The following morning, the tubes were centrifuged at room temperature for 5 min at 12,000×*g* to precipitate the pellet further. The sodium acetate/ethanol mixture was carefully removed with a pipette. The remaining DNA pellet was washed by slowly dripping in 3 ml of ice cold (− 20 °C) 70% ethanol and centrifuged at room temperature for 5 min at 12,000×*g*. The 70% ethanol was removed by pipette and the tubes were allowed to dry inverted on a fresh disposable lab wipe in a sterile, running hood for 1–1.5 h. Once the ethanol had completely evaporated and the pellets had dried, they were rehydrated with 25 µl of tris–EDTA (TE) buffer for 1–2 h.

#### DNA assessment

The DNA concentrations of the samples were measured in duplicate using an Epoch™ microplate spectrophotometer (Biotek, Winooski, VT, US) (Table 1). The DNA quality was assessed using the gDNA ScreenTape assay (PN # 5067-5365, Agilent) for the 2200 TapeStation system (Agilent) on a subsample (n = 7) of the 23 samples following manufacturer’s protocols. The assay provides a DNA integrity number (DIN) to score the DNA in the sample determined by the bp size of the product relative to the assay’s ladder and the peak height of the electropherogram produced and analyzed by the proprietary software [18]. A DIN of 10 represents highly intact gDNA and a DIN of 1 represents very degraded gDNA (Gassmann and McHoull, 2014). Our final use of the samples was a genome-wide genotyping by sequencing analysis (DArT: Diversity Arrays Technology, Bruce, Australia) for which the company requires high quality DNA and 500–800 ng of DNA per sample. Two microliters of each isolate with sufficiently high concentrations (~ 50 ng/µl) were run on a 0.8% agarose gel at ~ 90 V for an hour to test for DNA quality (fragmentation) before being shipped to the company.

**Table 1:** Quality measurements of DNA extracted from moose blood

Moose	DNA concentration (ng/µl)	DIN
Aa-1	337	
Aa-2	269	
Aa-3	265	
Aa-4	215	9.1
Aa-5	187	9.2
Aa-6	170	
Aa-7	162	
Aa-8	153	–
Aa-9	143	
Aa-10	141	
Aa-11	111	9.3
Aa-12	108	
Aa-13	92	9.4
Aa-14	80	
Aa-15	78	
Aa-16	74	
Aa-17	69	
Aa-18	51	
Aa-19	25	9.4
Aa-20	23	9.1
Aa-21	17	
Aa-22	15	
Aa-23	4	

The DIN is the DNA integrity number derived from the gDNA ScreenTape assay (Agilent) and is scaled from 1 to 10, with 1 being low integrity and 10 being the highest integrity

### Results

All samples yielded DNA with this protocol, with concentrations ranging from 4 to 337 ng/µl (mean 121 ng/µl) (Table 1). In comparison, other work (unpublished) where we have used a DNeasy Blood and Tissue Kit (Qiagen, Germantown, MD) to extract DNA from archived moose whole blood samples resulted in similar yields of DNA (N = 40, range = 40–307 ng/µl, mean = 69 ng/µl). The concentrations of the seven samples assessed on the TapeStation ranged from 23 to 215 ng/µl (Table 1). Six of the seven samples returned a DNA integrity number (DIN) greater than 9 with a range between 9.1 and 9.4 (Table 1). The remaining sample did not report a DIN as the sample concentration during the analysis was below the functional range of the assay, possibly due to improper sample preparation prior to the analysis.

**Fig. 1:**
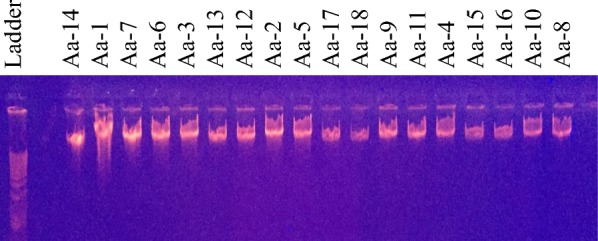
Gel electrophoresis results of 18 moose DNA isolates (extracted with modified PCI protocol)

Using this technique, 18 of the 23 samples resulted in a DNA yield > 50 ng/µl (Table 1) suitable for DArT analysis. The gel analysis of the genomic DNA indicated acceptable volumes of high molecular weight DNA (> 10 kbp) for all 18 of the DNA extracts (Fig. 1). The predominant band visible on the gel is mainly mitochondrial DNA, while the larger band closer to the loading well indicates intact nuclear DNA. These samples produced acceptable genotypes across 5809 loci within the population.

### Discussion

Blood draws are a minimally invasive, high quality, and high yield source for molecular materials (DNA, RNA, proteins, etc.) which can be used for a variety of research applications [19–21]. Our work has resulted in a useful protocol for extracting high quality genomic DNA from what may otherwise be discarded supernatant. Additionally, the method is comparable in effectiveness to available DNA extraction kits used for whole blood. The reagents used in the Tempus tube and subsequent RNA extraction protocol make the sample unsuitable for DNA extraction using such DNA extraction kits. However, using this protocol, the DNA isolated from the supernatant had sufficient integrity that it could be used for next generation sequencing downstream. Health assessments of moose, due their large size, relatively widespread occurrence on the landscape, and generally snowy conditions during the capture season, requires significant logistical preparation. This protocol allows us to minimize our impact on the animal by reducing the number of samples needed to support our research, and thereby reducing the processing time. Additionally, the use of products such as the Tempus blood tubes reduces the weight of supplies as no centrifuge or dry ice is needed to process/preserve the blood samples on site. By using one sample collection protocol for both DNA and RNA, the study maximizes efficiency. We recommend this simple procedure to maximize the yield of both RNA and DNA from blood samples.

## Limitations

This method assessed the suitability of using Tempus RNA blood tube supernatant remaining after RNA isolation for DNA isolation. Wherein we did observe effective isolation of DNA from these supernatants, we did not perform an isolation of DNA from unspent (RNA still present) Tempus tubes containing moose blood for comparison. This is because the blood samples collected from the moose populations in our study are hard to obtain, and the RNA collection is a priority for all the samples we collect.

## Abbreviations

CI: chloroform isoamyl alcohol
DArT: Diversity Arrays Technology
DIN: DNA integrity number
gDNA: genomic DNA
PBS: phosphate buffered serum
PCI: phenol chloroform isoamyl alcohol
TE: tris–EDTA
